# Localizing True Brain Interactions from EEG and MEG Data with Subspace Methods and Modified Beamformers

**DOI:** 10.1155/2012/402341

**Published:** 2012-06-27

**Authors:** Forooz Shahbazi Avarvand, Arne Ewald, Guido Nolte

**Affiliations:** ^1^IDA Group, Fraunhofer Institute FIRST, Kekuléstraße 7, 12489 Berlin, Germany; ^2^Department of Computer Science, Faculty of Mathematics and Natural Sciences II, Humboldt-Universitaet zu Berlin, Rudower Chausee 25, 10099 Berlin, Germany; ^3^Machine Learning Group, Berlin Institute of Technology, Franklinstr 28/29, 10587 Berlin, Germany; ^4^NIRx Medizintechnik GmbH, Baumbachstraße 17, 13189 Berlin, Germany; ^5^Department of Neurophysiology and Pathophysiology, University Medical Center Hamburg-Eppendorf, Martinistraße 52, 20246 Hamburg, Germany

## Abstract

To address the problem of mixing in EEG or MEG connectivity analysis we exploit that noninteracting brain sources do not contribute systematically to the imaginary part of the cross-spectrum. Firstly, we propose to apply the existing subspace method “RAP-MUSIC” to the subspace found from the dominant singular vectors of the imaginary part of the cross-spectrum rather than to the conventionally used covariance matrix. Secondly, to estimate the specific sources interacting with each other, we use a modified LCMV-beamformer approach in which the source direction for each voxel was determined by maximizing the imaginary coherence with respect to a given reference. These two methods are applicable in this form only if the number of interacting sources is even, because odd-dimensional subspaces collapse to even-dimensional ones. Simulations show that (a) RAP-MUSIC based on the imaginary part of the cross-spectrum accurately finds the correct source locations, that (b) conventional RAP-MUSIC fails to do so since it is highly influenced by noninteracting sources, and that (c) the second method correctly identifies those sources which are interacting with the reference. The methods are also applied to real data for a motor paradigm, resulting in the localization of four interacting sources presumably in sensory-motor areas.

## 1. Introduction

Electroencephalography (EEG) and magnetoencephalography (MEG) are noninvasive measurements of brain activity with an excellent temporal resolution in the order of milliseconds but poor spatial resolution. In the past decades the main focus was the analysis of event-related potentials, that is, the average brain response to a given stimulus. More recently, the variability of brain activity and especially its interpretation as signatures from the brain as a dynamical network has attracted many researchers [1, 2]. A specific expression of variability is the occurrence of neural oscillations which are hypothesized to be a mechanism of functional communication within the brain [3–6]. A large variety of methods exist to identify interactions of rhythmic activity, including coherence [7], AR modeling [8], Granger causality [9], and methods based on phase couplings [10].

The most serious problem in the interpretation of EEG or MEG in terms of brain interaction arises from the poor spatial resolution. First of all, at the sensor level it is not clear whether a functional relationship between sensors reflects an interaction between two different neural populations or is due to the mixing of sources into sensors. Furthermore, since the inverse problem, that is,the calculation of brain activity from EEG or MEG data, has no unique solution, any estimate is error prone: also estimated source activities are in fact a largely unknown mixture of the true activities. While this problem, usually termed “artifact of volume conduction,” is well known since a long time [11], it is increasingly addressed lately [12–19]. One major result is that also sophisticated and popular inverse methods like beamformers may produce substantial misinterpretations of the results [18].

To overcome the problem of volume conduction it was suggested to study the imaginary part of coherency [20] because nonvanishing values of that quantity can only be explained by true interactions. Imaginary part of coherency has been used for the estimation of functional connectivity inside the brain in several studies [21–23]. On the sensor level, this allows to establish the presence of brain interactions but only little can be said about the origins of the interactions inside the brain. Analysis on sensor level was further pursued in [24] where a method was proposed to separate pairs of interacting sources from each other. The results are two-dimensional subspaces which contain the topographies, that is the electric potentials or magnetic fields, for each pair of sources. A further decomposition into the topographies of the individual sources is not possible without making spatial assumptions about the sources. In [17] such a decomposition was performed assuming that respective source distributions, estimated as minimum L2-norm solutions, have minimal spatial overlap. The problem here is that such minimum norm solutions are extremely blurred, and even if the unknown true sources are not overlapping the estimates may have substantial spatial overlap.

In this paper we propose a more natural approach to deal with the case when the outcomes of a sensor level approach are subspaces rather than topographies of individual sources. In fact, subspace methods like the Multiple Signal Classification (MUSIC) and variants of it to be discussed below are designed to find sources which explain subspaces by dipolar sources [25–27]. Such methods are typically applied on low-rank approximations of covariance matrices and work optimally if all sources are independent of each other. To localize interacting sources, we here suggest to simply apply the subspace methods to low-rank approximations of the imaginary part of the cross-spectrum. We show next that this method correctly estimates the source locations also in the presence of strong background noise.

The second question we address is how to estimate with which other source each of the found sources is interacting. This analysis will be based on LCMV beamforming, which is a popular inverse method to analyze EEG or MEG data [12, 28]. Similarly to localization using MUSIC, we will adapt the beamformer to be most sensitive to interactions rather than being sensitive to strong power.

This paper is organized as follows. In Section 2 we will explain the mathematical background on the imaginary part of the cross-spectrum, subspace methods, and on beamformers. In Section 3 we will present our modifications of the RAP-MUSIC approach and of beamformers in order to study interacting brain sources, and in Section 4 we will demonstrate the performance using simulations of two and four interacting sources in the presence of background noise of various strengths as well as real data. A conclusion is given in Section 5.

## 2. Background

### 2.1. MUSIC and RAP-MUSIC

Multiple Signal Classification (MUSIC) is a localization method based on dominant subspaces spanned by the vector structure of the data [25]. The general procedure is to divide the vector space of the data into a signal subspace and a noise-only subspace which is orthogonal to the signal subspace. The algorithm is used for acoustic imaging [29, 30] and for the analysis of electrophysiological recordings of brain activity [26, 27]. It finds the source locations as those for which the principle angle between the noise subspace and the forward model of the source is maximum or, equivalently, for which the principle angle between the signal subspace and the forward model is minimal. In a nutshell, the MUSIC algorithm scans all possible source locations and estimates whether a source at each location is consistent with the measured data explicitly including the possibility that several sources are simultaneously active and in general not independent of each other.

We will at first consider the case of fixed dipole orientations. For MUSIC, a subspace of the signal is determined as the space spanned by the set of eigenvectors corresponding to the *P* largest eigenvalues of the covariance matrix of the data *C*, which itself has usually full rank for noisy data. *P* is the (assumed) number of sources, and it is assumed to be substantially smaller than the number of electrodes. (The true number of sources is in general unknown and it is advisable to choose *P* rather too large than too small.)

We denote the forward model of the dipole at location *q*_*i*_ as *L*_*M*×1_, where *M* is the number of electrodes. In order to estimate the consistency between the forward model of a given grid point and the subspace, the angle between them is calculated as

(1) $$\begin{array}{c} \cos^{2}\theta \left(L,\phi \right)=\frac{L^{T}\phi \phi^{T}L}{L^{T}L}, \end{array}$$

where (·)^*T*^ denotes transpose, and *ϕ* is the matrix of the *P* largest eigenvalues of the covariance matrix of the data. We note that formulations using angles between model and noise subspace are formally equivalent. Formulations using signal subspace only are computationally more efficient since the dimensionality of the signal subspace is lower compared to the dimensionality of the noise subspace.

The angle *θ* is calculated in all the grid points and the forward model corresponding to the minimum angle is estimated as the dipole pattern. If, as in EEG or MEG, for each grid point several forward solutions exist, corresponding to three different dipole orientations, the source orientation corresponding to the largest value of cos⁡*θ* is chosen.

The main disadvantage of MUSIC is that finding several maxima is difficult when the number of sources increases [27]. As a remedy, several modifications of MUSIC are proposed which are based on the idea of localizing the sources sequentially [27, 31–33]. One of the variants proposed by Mosher and Leahy [27] is a modification called Recursively Applied and Projected (RAP)-MUSIC. Here, instead of searching simultaneously for several local maxima, only global maxima are determined iteratively. In order to find the next source location, the subspace is updated by projecting out the previously found topographies and then the maximization is repeated.

To be explicit, let *L*_*k*_ for *k* = 1 ⋯ *n* − 1 be the set of patterns of the *n* − 1 previously found sources. In order to find the location of the *n*th source, the new subspace is defined by removing the patterns both from the forward models and the subspace estimation. The projection matrix for the *n*th source estimation reads

(2) $$\begin{array}{c} P=I-A{\left(A^{T}A\right)}^{-1}A^{T}, \end{array}$$

where *A* = [*L*_1_ *L*_2_ ⋯ *L*_*n*−1_] is the matrix containing as columns all the previously found dipole patterns.

Similarly to the first MUSIC scan, the angle between the forward model at each grid point and the subspace is calculated while the forward models and the subspace are updated by projecting out the previous source patterns. Therefore

(3) $$\begin{array}{c} \cos^{2}\theta \left(L_{P},\phi_{P}\right)=\frac{L_{P}^{T}\phi_{P}\phi_{P}^{T}L_{P}}{L_{P}^{T}L_{P}}, \end{array}$$

where *L*_*P*_ = *PL* and Φ_*P*_ = ortho(*P*Φ) where ortho(*E*) orthonormalizes the columns of a matrix *E*.

The algorithm performs as many iterations as the predefined number of sources.

For unknown dipole orientations an optimization over orientation is included in the calculation of the angle *θ*. Then

(4) $$\begin{array}{c} L=\hat{L}\alpha , \end{array}$$

where $\hat{L}$ is an *N* × 3 matrix containing as columns the topographies of dipoles in *x*-, *y*- and *z*-direction and *α* is a 3 × 1 vector. Then the angle is given as

(5) $$\begin{array}{c} \cos^{2}\theta \left(L_{P},\phi_{P}\right)=\underset{\alpha }{\max}\frac{\alpha^{T}{\hat{L}}_{P}^{T}\phi_{P}\phi_{P}^{T}{\hat{L}}_{P}\alpha }{\alpha^{T}{\hat{L}}_{P}^{T}{\hat{L}}_{P}\alpha }, \end{array}$$

with ${\hat{L}}_{P}=P\hat{L}$. The maximization can be done analytically, and *α* is given by the eigenvector corresponding to the maximum eigenvalue of

(6) $$\begin{array}{c} D\equiv {\left({\hat{L}}_{P}^{T}{\hat{L}}_{P}\right)}^{-1}{\hat{L}}_{P}^{T}\phi_{P}\phi_{P}^{T}{\hat{L}}_{P}. \end{array}$$

Note that the RAP-MUSIC search results both in location and orientation of the sources.

### 2.2. Imaginary Part of Cross-Spectrum

A covariance matrix is a measure of linear coupling between two signals in the time domain. The analogue in the Fourier domain is the complex valued cross-spectrum which reflects the linear coupling of the signals for all frequencies. Due to the artifacts of volume conduction, it is not always easy to differentiate between the real connectivities and the ones caused by volume conduction. Nolte et al. [20] suggest that the imaginary part of coherency, which is in fact the normalized imaginary part of cross-spectrum, is a measure robust to artifacts of volume conduction in the sense that a nonvanishing imaginary part cannot be explained by independent sources regardless of the number of sources and how they are mapped into sensors provided that this mapping is essentially instantaneous which is in fact an excellent approximation for frequencies below 1 KHz [34].

Coherency between two EEG channels *i* and *j* is defined as

(7) $$\begin{array}{c} C_{ij}\left(f\right)=\frac{S_{ij}\left(f\right)}{{\left(S_{ii}\left(f\right)S_{jj}\left(f\right)\right)}^{1/2}}, \end{array}$$

where *S*_*ij*_(*f*) is the cross-spectrum of the two channels at frequency *f* and is defined as

(8) $$\begin{array}{c} S_{ij}\left(f\right)=\langle x_{i}\left(f\right)x_{j}^{*}\left(f\right)\rangle , \end{array}$$

where *x*_*i*_(*f*) and *x*_*j*_(*f*) are the (complex) Fourier transformations of the time series of *x*_*i*_(*t*) and *x*_*j*_(*t*) of channels *i* and *j*, respectively, 〈·〉 is the expectation value, and ∗ is complex conjugation. In practice, the expectation value is obtained by averaging over a large number of epochs. *S*_*ii*_(*f*) and *S*_*jj*_(*f*) are the autospectra of the signals at channels *i* and *j*, respectively.

### 2.3. Beamformers

The goal of beamforming is to estimate the time course *s*(*t*) of a dipole at a specific location in the brain as accurate as possible. To achieve that goal, sensor data are linearly combined such that the (presumed) activity of other sources is minimized [35]. We here recall shortly the basic procedure.

If the location where we want to calculate the time course is *q*_*i*_ and the activity of the dipole at this location is *s*_*i*_(*t*), the data **X**(*t*) measured with EEG electrodes is the superposition of *N* dipoles at sampling time *t*:

(9) $$\begin{array}{c} X\left(t\right)=\sum_{i=1}^{N}g_{i}s_{i}\left(t\right)+n\left(t\right)‍, \end{array}$$

where **g**_*i*_ is the forward model for a source at location *q*_*i*_ with given orientation. The vector **g**_*i*_ is of size *M* × 1, where *M* is the number of electrodes and *n*(*t*) is additive noise which is assumed here to arise from noninteracting sources. A beamformer is a spatial filter constructed to (a) pass the signal from the source of interest with unit magnitude and (b) to minimize total power. If the source of interest is independent of all other sources (called background), power values of source of interest and background are additive. Therefore, minimizing total power is equivalent to minimizing the power of the background and hence to maximizing signal-to-noise ratio. At a specific location inside the brain we estimate the signals *y*_*j*_(*t*), for *j* = 1,2, 3, corresponding to the source component in *x*-, *y*-, and *z*-direction, as

(10) $$\begin{array}{c} y_{j}\left(t\right)=W_{j}^{T}X\left(t\right). \end{array}$$

If we denote the topography of the source in direction *j* as **g**_*j*_ the filter weights *W*_*j*_ are chosen to satisfy the following constraint:

(11) $$\begin{array}{c} \min_{W_{j}}\left(W_{j}^{T}CW_{j}\right) \end{array}$$

(12) $$\begin{array}{c} \text{subject to }W_{j}^{T}g_{j}=1, \end{array}$$

where the matrix *C* is the covariance matrix of the measured data in time domain or the cross-spectrum matrix in frequency domain. The optimization is solved by

(13) $$\begin{array}{c} W_{j}=C^{-1}g_{j}{\left[g_{j}^{T}C^{-1}g_{j}\right]}^{-1}. \end{array}$$

So far, all dipole components were calculated, and such a beamformer is called “vector beamformer.” To specify the direction at each grid point, it is a common approach to maximize the power. We only mention this without going into details, because we are interested in observing interacting and not necessarily strong sources. The respective choice of orientation will be explained in Section 3.

One point which should be considered in beamforming is the correlation between the sources. Due to the presence of correlated sources, the estimated variance of the source of interest is significantly less than the true value. Therefore, a modified version of LCMV called Nulling Beamformer [12, 36] was suggested forcing an additional nulling constraint in order to make sure that the influence of the sources at specific other locations and orientations is suppressed. We recall the procedure for a set of sources with given orientation. Combining the nulling constraint with the unit gain condition (12) in LCMV results in

(14) $$\begin{array}{c} W_{i}^{T}G=f_{i}^{T}, \end{array}$$

where *G* = [**g**_1_,…, **g**_*N*_] contains as columns the topographies of *N* sources and

(15) $$\begin{array}{c} f_{i}={\left[0\cdots 010\cdots 0\right]}^{T}, \end{array}$$

is a vector whose *i*th element is one and the rest are zero. Solving the equation, using Lagrange multipliers, results in

(16) $$\begin{array}{c} W_{i}=C^{-1}G{\left[G^{T}C^{-1}G\right]}^{-1}f_{i}. \end{array}$$

The obtained nulling beamformer gain has a unit gain at the location of interest, zero gains at a small set of given locations other than the location of interest and minimizes the power for the *i*th source.

We finally note that a vector beamformer is often formulated as a nulling beamformer for which for each dipole location and direction *j* both other orthogonal directions were nulled out.

An LCMV beamformer is really a two-step procedure with two different rationals. In the first step, spatial filters are designed to estimate brain activity for each location in the brain as clean as possible. This step is not a localization approach. The localization is done in the second step by defining the most interesting sources as those which have strongest power. Below, we will use only the first step of the beamformer formulation because we are interested only in interacting sources which are not necessarily the ones with strongest power.

## 3. New Methods

### 3.1. Getting Subspaces from Imaginary Part of Cross-Spectrum (CS)

 The standard way to define the subspace of the data used for the MUSIC algorithm, as we discussed in Section 2.1, is to calculate the eigenvectors of the covariance matrix of the data. We suggest to replace the covariance matrix by the imaginary part of the cross-spectrum of the data at a specific frequency in RAP-MUSIC. As we discussed in Section 2.2, the imaginary part of the cross-spectrum is inconsistent with noninteracting sources. Since we are interested in localizing the interacting sources, we defined the subspace of the data based on the imaginary part of the cross-spectrum just to make sure that noninteracting sources like noise do not appear in localization results.

### 3.2. Maximizing Imaginary Coherence in Subspaces

According to the definition, coherency between two EEG channels *i* and *j* is equivalent to the complex valued cross-spectrum normalized by the power in the channels. In order to calculate the coherency between the source location *i* and any other grid point in the brain, the signals originating from these locations will be calculated using a beamformer. The moment of a dipole at location *j* is therefore calculated as

(17) $$\begin{array}{c} y\left(f\right)=AX\left(f\right), \end{array}$$

where **X**(*f*) is the Fourier transform of the data at frequency *f* and filter weights,*A*, are calculated using either LCMV beamformer or nulling beamformer. Let *G* = [**g**_1_,…, **g**_*N*_] be the matrix of dipole patterns of the dipoles estimated at the source locations by RAP-MUSIC then the activity of the source, *s*(*f*), in the frequency domain at the *i*th location is

(18) $$\begin{array}{c} s\left(f\right)=V_{i}^{T}X\left(f\right),V_{i}={\left(C_{R}\right)}^{-1}G{\left[G^{T}{\left(C_{R}\right)}^{-1}G\right]}^{-1}f_{i}. \end{array}$$

The filter weights at each source location **V**_*i*_ are calculated using nulling beamformer weights in (16) and the vector **f**_*i*_ is defined in (16). The filter weights could also be estimated using LCMV approach in (13) but in order to reduce the interaction of other sources in the estimation of the time course of the source of interest using nulling beamformer is preferred. The matrix *C* in (16) and (13) is replaced by the real part of cross-spectrum matrix of the data, *C*_*R*_, in order to have real-valued weights instead of complex ones.

In a similar approach, the activity of each grid point is calculated using (17) where the filter weights, *A*, are calculated based on LCMV beamforming weights in (13) in directions *x*, *y*, and *z*. In the classical LCMV beamformer, the direction of the dipole is chosen as the direction which maximizes the power of the signal at the corresponding location but in a new approach, we suggest to choose the direction of the dipole not based on the maximum power but on the maximum imaginary part of coherency between a reference and the dipole of interest.

Assuming *z*(*f*) is the moment of the dipole at frequency *f* at location *j* in the direction of maximum coherency with location *i* (the seed location) then,

(19) $$\begin{array}{c} z\left(f\right)=\alpha^{T}AX\left(f\right), \end{array}$$

where *α* is the vector of size 3 × 1 which gives us the (yet unknown) direction in which the imaginary part of coherency is maximum.

The cross-spectrum between the estimated source at location *i*, *s*(*f*), and the activity in direction of maximum coherency at location *j*, *z*(*f*), is defined as

(20) $$\begin{array}{c} \langle s\left(f\right)z^{*}\left(f\right)\rangle =V_{i}^{T}\langle X\left(f\right)X^{H}\left(f\right)\rangle A^{T}\alpha , \end{array}$$

where 〈·〉 denotes the expectation value and ∗ is the complex conjugation. The autospectrum of the signal *s* at location *i* is defined as

(21) $$\begin{array}{c} \langle s\left(f\right)s^{*}\left(f\right)\rangle =V_{i}^{T}\langle X\left(f\right)X^{H}\left(f\right)\rangle V_{i}, \end{array}$$

where 〈**X**(*f*)**X** ^*H*^(*f*)〉 is equal to the cross-spectrum of the data. Similarly, the cross-spectrum of *z*(*f*) at location *j* is defined as

(22) $$\begin{array}{c} \langle z\left(f\right)z^{*}\left(f\right)\rangle =\alpha^{T}A\langle X\left(f\right)X^{H}\left(f\right)\rangle A^{T}\alpha . \end{array}$$

The imaginary part of coherency then reads

(23) f(α)=〈s(f)z∗(f)〉〈s(f)s∗(f)〉〈z(f)z∗(f)〉=ViTCIATα(ViTCVi)1/2(αTACATα)1/2

where *C*_*I*_ denotes the imaginary part of cross-spectrum of **X**(*f*). Let us rename (**V**_*i*_^*T*^ *C* **V**_*i*_) as *D*_1_, (*α* ^*T*^ *ACA* ^*T*^ *α*) as *D*_2_, and **V**_*i*_^*T*^ *C*_*I*_ *A* ^*T*^ *α* as *N*. In order to maximize *f*(*α*), we set the derivative of *f*(*α*) to zero:

(24) $$\begin{array}{c} \frac{\partial f}{\partial \alpha }=\frac{{\left(V_{i}^{T}C_{I}A^{T}\right)}^{T}}{D_{1}^{1/2}D_{2}^{1/2}}-\frac{1}{2}\frac{N}{D_{1}^{1/2}D_{2}^{1/2}}\frac{\partial }{\partial \alpha }\left(\alpha^{T}AC_{R}A^{T}\alpha \right)=0, \end{array}$$

where *C*_*R*_ is the real part of *C*. Solving the equation results in

(25) $$\begin{array}{c} \alpha ={\left(AC_{R}A^{T}\right)}^{-1}AC_{I}V_{i}. \end{array}$$

Substituting *α* in (23) gives us the maximum of the imaginary part of coherency at frequency *f*. We used the above maximization of coherency after applying the RAP-MUSIC algorithm to the data in order to study the interactions between the localized sources.

## 4. Results

### 4.1. Simulations

In this Section, we present the simulations in which we compared the RAP-MUSIC results in the case the subspace of the data is defined with the largest eigenvalues of real part of the cross-spectrum to the case that the subspace is defined based on the imaginary part of the cross-spectrum. We also demonstrate the results of finding the interaction between the sources after being localized by RAP-MUSIC.

In the first simulation, two interacting sources are produced at 10 Hz with the sampling frequency of 100 Hz. The interaction was simulated simply as a delay: if *x*_1_(*t*) is the signal of the first source, white noise narrowband filtered at 10 Hz, then the signal of the second source reads *x*_2_(*t*) = *x*_1_(*t* − *τ*) with the delay set as *τ* = 20 ms. The total length of the data is 300 sec and is divided into segments of 100 samples each. Each segment has 50 samples overlapping with the previous segment. Additional noise resembling the real brain noise is added to the data. This is done by simulating independent white noise source distributed evenly across the entire brain. The noise was scaled such that the power at 10 Hz at the strongest signal channel was equal for noise and signal of interest.

For the forward solution, calculated using expansions of the electric lead field [37], we used a realistic head model based on a segmented head model taken from the program CURRY. In this simulation, the dimension of the subspace, that is, *P*, was chosen to be equal to two.

**Figure 1:**
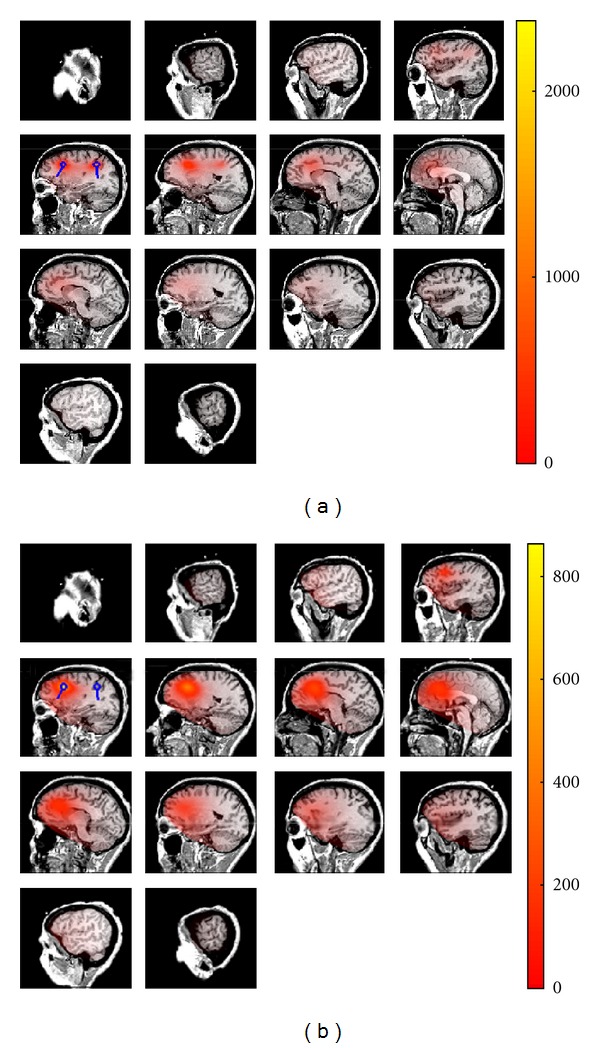
Estimated locations of the sources using RAP-MUSIC based on the imaginary part of cross-spectrum. True locations of the sources are shown as blue dipoles and the estimated locations are shown using the heat map. (a) represents the sources after applying the first iteration of RAP-MUSIC and (b) shows the source location after projecting out the global maximum of (1) in the previous iteration.

**Figure 2:**
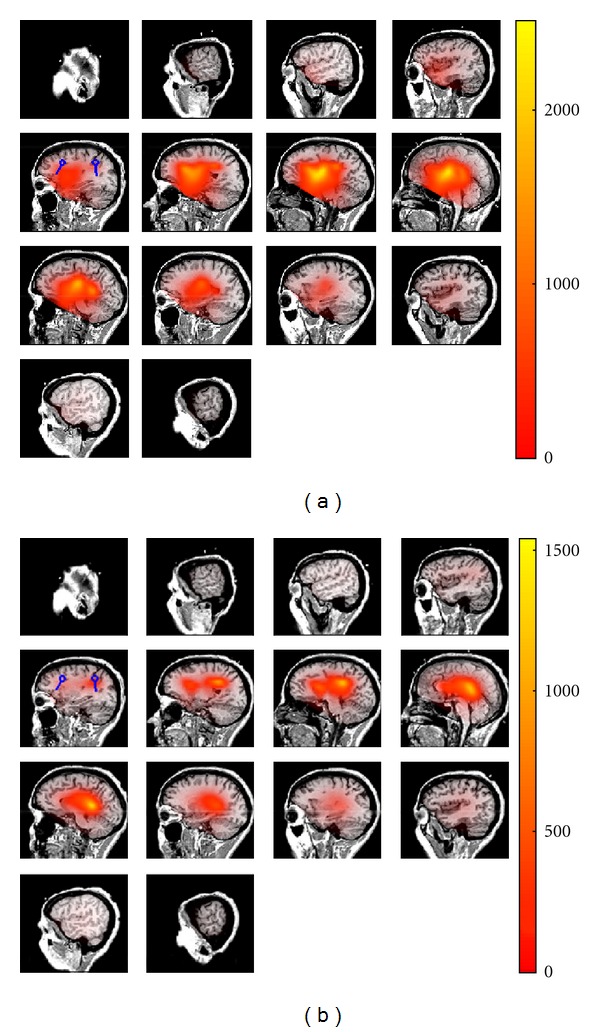
Estimated locations of the sources using RAP-MUSIC based on the real part of cross-spectrum. True locations of the sources are shown as blue dipoles and the estimated locations are shown using the heat map. (a) represents the sources after applying the first iteration of RAP-MUSIC and (b) shows the source location after projecting out the global maximum of (1) in the previous iteration.

Figures 1 and 2 are illustrations of the estimated source locations using the real and imaginary parts of cross-spectrum. For each voxel, the result of 1/(1−|cos⁡*θ*|) is color coded. The blue circles in the Figures represent the true locations of the simulated sources.  Figure 1 shows the two estimated sources using imaginary part of the cross-spectrum. In the first RAP-MUSIC step, both of the two sources are localized simultaneously. In the next step the first source is projected out and only one of the sources has remained. Comparing the results in Figure 1 and RAP-MUISC based on the real part of cross-spectrum in Figure 2 shows that the localization accuracy increases massively when the imaginary part is used to estimate the sources. In fact, we can see source location estimations in Figure 2 which do not fit our true locations. These estimations are the locations where noninteracting sources, noise in this case, are dominant.

**Figure 3:**
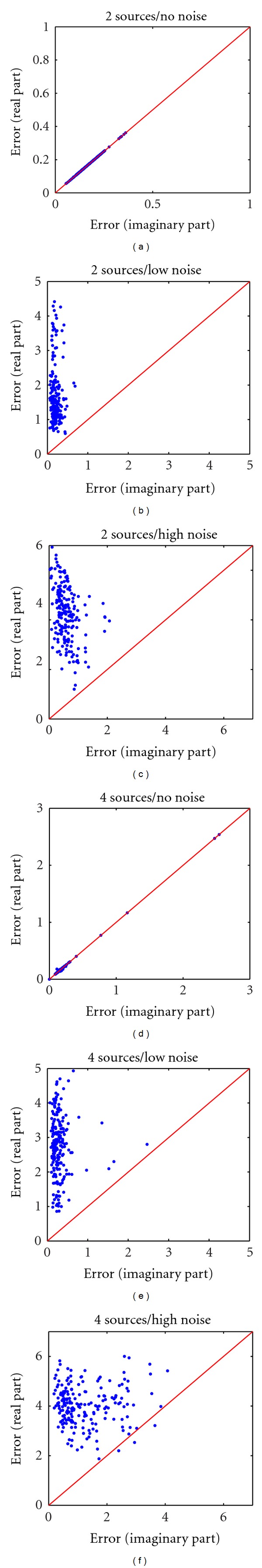
Estimation error in RAP-MUSIC based on the real and imaginary part of the cross-spectrum. (a), (b), and (c) show the simulations of two interacting sources in 200 independent simulation cases in random locations. (d), (e), and (f) represent four sources containing two couples of interacting sources simulated in 200 independent simulation cases in random locations. The simulations were done for three different noise levels as explained in the text and as indicated in the figure.

Figure 3 shows the estimation error for two and four interacting sources, which are located at random positions inside the head and had random orientations, for 200 independent simulations. We did the simulations with three different noise levels: (a) no noise, (b) low noise corresponding to equal power of noise and signal of interest at 10 Hz averaged over all channels, and (c) high noise corresponding to equal power of noise and signal of interest at 10 Hz at the channel with largest power of the signal of interest.

On the *x*-axis the localization error of RAP-MUSIC based on the imaginary part of cross-spectrum is shown and the *y*-axis represents the estimation error resulting from the real part of cross-spectrum. To identify estimated source locations with true ones we calculated the mean distance across all permutations and chose the one which minimized this mean. The error localizations based on the real part of the cross-spectrum are considerably larger than for the imaginary part of cross-spectrum. In fact, by considering the imaginary part of cross-spectrum, we reduce the effect of the noninteracting noise sources on the localization of the interacting sources.

**Figure 4:**
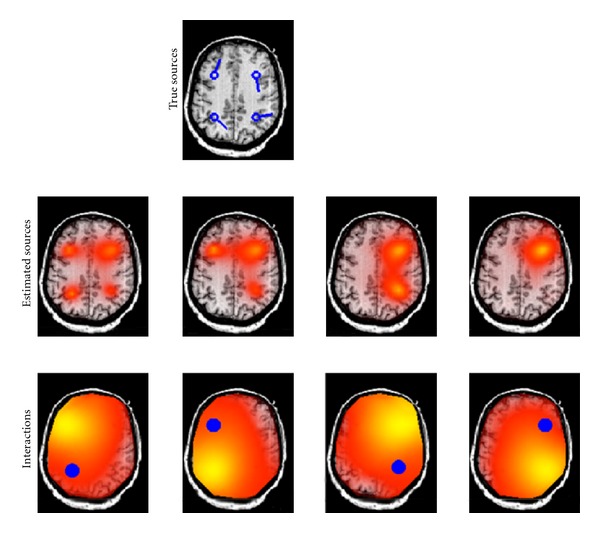
Four interacting dipoles are simulated. Each two sources in one hemisphere are interacting with each other. In the top figure, the location of true sources are shown. In the middle row, estimated source locations using RAP-MUSIC based on the imaginary part of cross-spectrum are demonstrated and in the bottom row the area which is interacting with each of the corresponding sources (visualized as blue dots) is demonstrated.

In order to study the connectivity of the sources, we proceeded the simulations by applying the nulling beamformer to the EEG data and maximizing the imaginary part of coherency between the estimated source locations obtained from RAP-MUSIC and all other grid points. We demonstrate a typical outcome for a case consisting of four dipoles, two on the left and two on the right hemisphere with interactions within but not across hemispheres and a high noise level (Figure 4). For illustrative purposes, all dipoles were chosen to be in one axial plane, and, although the reconstruction was done in the entire brain, we show only this plane. The results fulfilled our expectations in the way that the highest imaginary part of coherency occurred almost at the same position as the true interacting source positions.

### 4.2. Real Data

We applied RAP-MUSIC to the real data measured during the imagined hand movement [38] in order to localize four interacting sources as well as their interactions. The cross-spectrum has a dominating alpha rhythm at 10 Hz which is not induced by the task but is considered to be an ongoing activity present at the eyes-open condition as well. The data contains central alpha also at 10 Hz due to event-related synchronization which in this case is induced by the absence of the foot movement which has been the task in nonanalyzed trials. The number of EEG channels is equal to 118 and the number of trials to 70 each with the duration of 3.5 s. The cross-spectrum at 10 Hz is measured with the frequency resolution of 2 Hz. Channel locations were matched on a realistic standard head model taken from the program CURRY (Neuroscan, Hamburg, Germany). 

**Figure 5:**
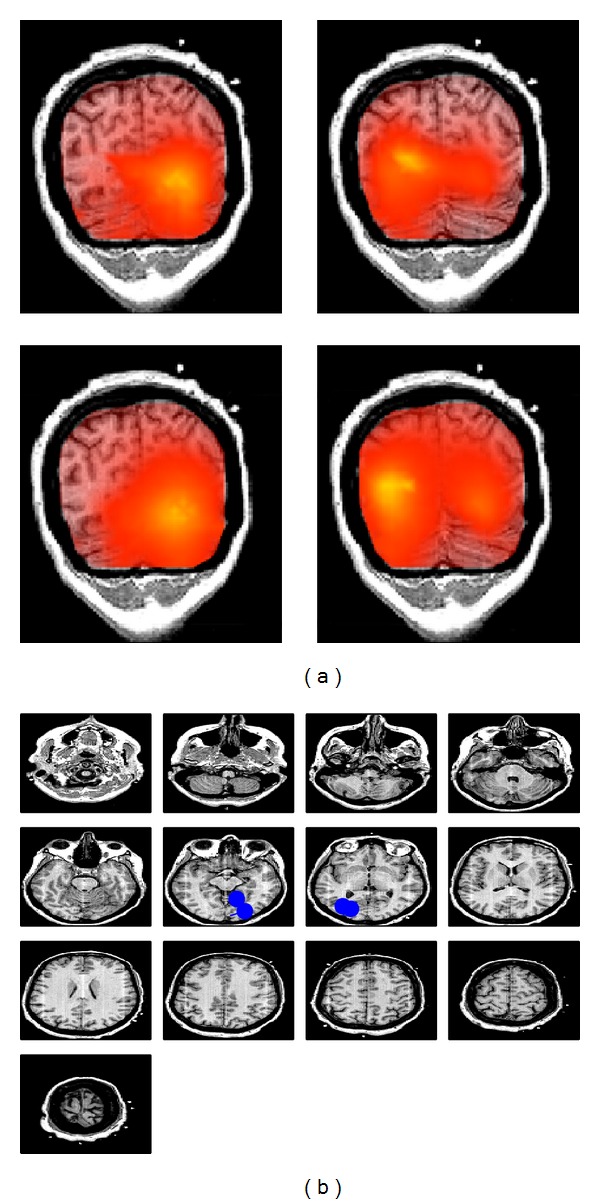
(a) RAP-MUSIC applied to the real data to localize 4 interacting sources. Each panel in (a) represents an iteration in RAP-MUSIC starting from the top left panel, then top right and so forth (b) The blue dipoles represent the final estimated source locations after all iterations.

**Figure 6:**
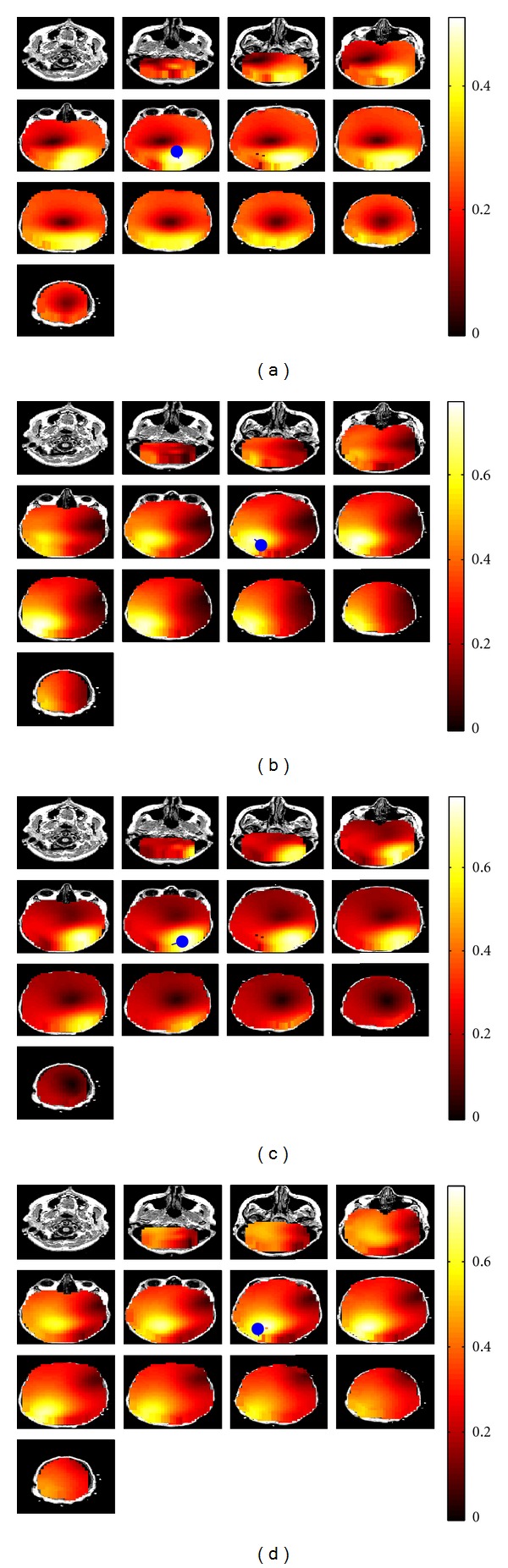
Localization of the interaction between four sources using the maximization of imaginary part of coherency. The blue dipoles represent the estimated location of each source which is taken as the reference and the heat map represents the area that is interacting with the corresponding reference location.

In order to apply RAP-MUSIC based on the imaginary part of cross-spectrum we set the dimension of the subspace equal to four. The source localization resulting in Figure 5 shows two sources in occipital lobe representing the alpha rythm. Two other sources are more close to the motor cortex which represent the absence of imagined foot movement. To study the interaction between the sources using beamformers and maximization of imaginary coherency described in Section 3, the imaginary part of coherency is maximized between each reference location found in RAP-MUSIC and each grid point. According to the results in Figure 6, the dipoles in each lobe are interacting locally with each other.

## 5. Conclusion

We adapted two well-established methods, the RAP-MUSIC approach and the LCMV beamformer approach, to localize and characterize interacting brain sources from rhythmic EEG or MEG data. To study brain interactions robust to artifacts of volume conduction, it is convenient to analyze the imaginary part of the cross-spectrum which is unbiased by noninteracting sources. In contrast to covariance matrices or complex cross-spectra the imaginary part, being antisymmetric, is necessarily degenerate: all singular values occur in pairs, and, for example, a singular value decomposition is not capable to extract the topographies of the individual sources in sensor space even if the true topographies are orthogonal. This is a principle limitation when analyzing interacting sources where dynamical assumptions like statistical independence, as is done for ICA, are inconsistent with the object which is studied. Rather than individual topographies results are naturally subspaces, for which subspace methods are ideal candidates to find the respective sources. In simulations we have shown that RAP-MUSIC, applied on subspaces given by imaginary parts of cross-spectra, properly recovers source locations also in the presence of strong correlated background noise, which was assumed to be generated by noninteracting sources.

This was shown for two and four sources, but not for three. The case of having an odd number of sources differs substantially from the case of an even number. The rank of an antisymmetric matrix is always even and we can only observe in the data an unknown two-dimensional projection of the three-dimensional subspace spanned by all three topographies. The presented RAP-MUSIC approach is in general not capable to localize sources properly in that case. This problem will be addressed in future work.

To estimate the interaction pattern we adapted the well-known LCMV beamformer to our needs. In “classical” beamformer algorithms the orientation for a given dipole is chosen as the one which maximizes the power in that brain voxel such that the solution picks the strongest source. This was replaced by choosing the direction to maximize the imaginary part of coherency between that voxel and a given reference. To avoid confounding effects by assigning interactions to wrong voxels we also chose to use the Nulling beamformer which sets additional constraints to explicitly exclude contributions from a given set of topographies. This set was defined in terms of the pair of voxels of which the interaction is calculated but a generalization to include other sources is straight forward.

An important advantage of studying the imaginary part of cross-spectra to localize interacting brain sources is that it is applicable without any modification also to differences of cross-spectra estimated, for example, in two different measurement conditions. An analogous property for the characterization of the interaction, that is, the question which source is interacting with which other, is not possible within the proposed scheme because coherence loses its meaning and is eventually even ill-defined when cross-spectra are normalized with power differences rather than powers. How to characterize interaction from difference of cross-spectra only will be addressed in future work.
